# Keystone Veterinary Medical Association

**Published:** 1892-01

**Authors:** W. S. Kooker

**Affiliations:** Secretary


*Keystone Veterinary Medical Association.*—The regular monthly meeting of Keystone Veterinary Medical Association was held at the College of Physicians, Philadelphia, December 5th, 1891. The meeting was called to order by the President, W. Horace Hoskins. There answered roll call, Drs. Hoskins, Huidekoper, Weber, Kooker, Cullen, Webster, Eves, and Sellers. There were present as visitors, Dr. Thos. B. Rayner, Dr. H. B. McDowell, and a number of laymen. The application of Dr. H. B. McDowell, Middletown, Del., for membership was presented, duly signed and vouched for. Referred to the proper committee. Dr. R. S. Huidekoper gave an address on the origin of the eight races of horses and the development of artificial races, like the Standard bred trotter, the Hackney, the Cleveland Bay, French Coacher, etc. A vote of thanks was tendered Dr. Huidekoper for his able and instructive address.

Dr. W. Horace Hoskins reported the case of the stallion "Eurus," the property of A. J. Cassett, Esq. The horse had been subject to pains, resembling colic, for a period of two or three years, generally after exercise or hard work. For some time past he has had an unthrifty appearance, with great loss of flesh. He had an attack of colic, lasting two days, from which he died. Autopsy revealed a tumor attached to the muscles of the abdomen, weighing 40 pounds. Dr. Huidekoper said that several years ago he had treated Eurus for colic, and had been consulted in regard to attacks of colic which came on after a race. These attacks were irregular, and it had been impossible to assign any cause for them, as they were neither typical cases of the flatulent nor of the congestive form of the disease. The tumor now accounts for the origin. Undoubtedly, pain caused by the tumor may account for certain failures in his races, causing a "cramp" during the race. The tumor will be examined microscopically and reported on later. Dr. Hoskins, President, congratulated the Keystone Association upon the result of their agitation of the subject of meat inspection. The city authorities having appointed two Veterinarians to the position of inspectors of meats. On motion adjourned.

W. S. Kooker, *Secretary.*

